# Preoperative embolization in the treatment of patients with metastatic epidural spinal cord compression: A retrospective analysis

**DOI:** 10.3389/fonc.2022.1098182

**Published:** 2022-12-15

**Authors:** Bin Zhang, Haikuan Yu, Xiongwei Zhao, Xuyong Cao, Yuncen Cao, Xiaolin Shi, Zheng Wang, Yaosheng Liu

**Affiliations:** ^1^ Senior Department of Orthopedics, The Fourth Medical Center of PLA General Hospital, Beijing, China; ^2^ Department of Orthopedic Surgery, National Clinical Research Center for Orthopedics, Sports Medicine & Rehabilitation, Chinese PLA General Hospital, Beijing, China; ^3^ Chinese PLA Medical School, Beijing, China; ^4^ Department of Orthopedic Surgery, The Fifth Clinical Medical College of Anhui Medical University, Beijing, China; ^5^ Department of Orthopedic Surgery, The Fifth Medical Center of PLA General Hospital, Beijing, China; ^6^ Department of Orthopedic Surgery, The Second Affiliated Hospital of Zhejiang Chinese Medical University, Hangzhou, China

**Keywords:** decompressive surgery, metastatic epidural spinal cord compression, blood loss, prognosis, preoperative embolization

## Abstract

**Purpose:**

The purpose of the study was to assess the effectiveness and safety of preoperative embolization in the treatment of patients with metastatic epidural spinal cord compression (MESCC).

**Methods:**

A retrospective analysis of 138 MESCC patients who underwent decompressive surgery and spine stabilization was performed in a large teaching hospital. Among all enrolled patients, 46 patients were treated with preoperative embolization (the embolization group), whereas 92 patients did not (the control group). Patient’s baseline clinical characteristics, surgery-related characteristics, and postoperative neurological status, complications, and survival prognoses were collected and analyzed. Subgroup analysis was performed according to the degree of tumor vascularity between patients with and without preoperative embolization.

**Results:**

Patients with severe hypervascularity experienced more mean blood loss in the control group than in the embolization group, and this difference was statistically significant (P=0.02). The number of transfused packed red cells (PRC) showed a similar trend (P=0.01). However, for patients with mild and moderate hypervascularity, both blood loss and the number of PRC transfusion were comparable across the two groups. Regarding decompressive techniques, the embolization group (64.29%, 9/14) had a higher proportion of circumferential decompression in comparison to the control group (30.00%, 9/30) among patients with severe hypervascularity (P=0.03), whereas the rates were similar among patients with mild (P=0.45) and moderate (P=0.54) hypervascularity. In addition, no subgroup analysis revealed any statistically significant differences in operation time, postoperative functional recovery, postoperative complications, or survival outcome. Multivariate analysis showed that higher tumor vascularity (OR[odds ratio]=3.69, 95% CI [confident interval]: 1.30-10.43, P=0.01) and smaller extent of embolization (OR=4.16, 95% CI: 1.10-15.74, P=0.04) were significantly associated with more blood loss.

**Conclusions:**

Preoperative embolization is an effective and safe method in treating MESCC patients with severe hypervascular tumors in terms of intra-operative blood loss and surgical removal of metastatic tumors. Preoperative tumor vascularity and extent of embolization are independent risk factors for blood loss during surgery. This study implies that MESCC patients with severe hypervascular tumors should be advised to undergo preoperative embolization.

## Introduction

Metastatic epidural spinal cord compression (MESCC) is the secondary compression of the spinal cord due to cancer metastases to the spine or epidural space, and it can reduce the quality of life because of cancer-associated back and leg pain, neurological deficit, and loss of bladder and bowel continence (1, 2). The morbidity of this disease is about 5%–10% among patients with malignant tumors (1), and approximately one out of ten spine metastases patients will develop MESCC (3). Therapeutic standards for MESCC patients are not yet accessible. Decompressive surgery followed by postoperative irradiation is typically recommended among individuals who have progressive neurologic deficit, a generally healthy level of physical activity, and an anticipated survival period of longer than three months (4).

Nevertheless, intra-operative blood loss poses a great significant problem for MESCC patients undergoing decompressive surgery. Several publications have noted that that surgically treated patients with metastatic spinal illness experienced blood loss of 1,630 to 3,640 ml (5) and sometimes up to 10,000 ml of significant bleeding (6), and a meta-analysis revealed that the pooled mean blood loss was above 2100 ml (7). Allogeneic blood transfusion was necessary for those patients under these circumstances, but it has been shown to be linked to an increased risk of postoperative infection, delirium, venous thromboembolism, and even mortality (8, 9). Thus, it is an intriguing topic to discuss ways to assist patients and surgeons in minimizing blood loss and transfusion.

Preoperative embolization is a technique that was developed to lessen intra-operative blood loss, simplify the process of spine surgery, and make operation safer (10–12). This method was first made available in 1974 to treat spine tumors and lessen intra-operative blood loss (13). Nowadays, it is widely used in the treatment of a variety of spinal tumors (10–12). However, several investigations showed that intra-operative blood loss during the surgical excision of spinal tumors was not significantly affected by preoperative embolization (11, 14, 15). This might be the case since the amount of blood loss varied greatly according to histology and surgical methods (16). In particular, highly vascularized cancers such as renal and live carcinoma were relevant to a high risk of blood bleeding, and more invasive procedures like corpectomy resulted in a significantly high volume of blood loss in comparison to laminectomy (16). More recently, a number of studies with a small size sample reported that preoperative embolization for patients with hypervascular metastatic tumors was able to decrease intra-operative blood loss (17, 18), but inconsistent results still existed (19, 20). In addition, patients with non-hypervascular lesions did not experience the benefit of lowering blood loss (16). Additionally, these findings still require further verification (21).

Therefore, this study aimed to assess the effectiveness and safety of preoperative embolization in the treatment of MESCC patients. Intra-operative features such as blood loss, number of packed red cells (PRC), and surgical methods and postoperative outcome including complication, functional recovery, and survival outcome were thoroughly collected and compared in the study to evaluate the role of preoperative embolization in MESCC patients. This study speculated that preoperative embolization might be an efficient and safe method to reduce blood loss during surgery, which would make it easier to remove metastases.

## Patients and methods

### Inclusion criteria and exclusion criteria

This study retrospectively examined 138 MESCC patients underwent decompressive surgery and spine stabilization between January 2012 and December 2018. Patients were considered for analysis if they met the following criteria: (1) patients were diagnosed with metastatic spinal cord compression, (2) patients were treated with decompression and spine stabilization combined with or without preoperative embolization, and (3) patients presented at least one of symptoms listed below as a result of MESCC: a. severe back pain; b. sensory dysfunction; c. motor dysfunction; d. sphincter dysfunction. Patients were excluded for the analysis if they met any of the following criteria: (1) age less than 18 years, (2) MESCC due to primary spinal malignant tumor or intramedullary metastases, (3) prior spinal surgery treatment, (4) poor health precluding surgery (an expected lifespan of less than three months), or (5) uncorrectable coagulopathy or renal impairment. Patient’s flowchart is outlined in **Supplementary Figure 1**.

Patients included in the analysis were classified according to the presence of preoperative embolization, and there were the embolization group and the control group. Patients in the embolization group were treated with preoperative embolization, whereas patients in the control group did not receive preoperative embolization. The Ethics Committee Board of the Fourth Medical Center of PLA General Hospital and waived the informed consent from patients since all data were retrospective in nature, and the study was conducted in accordance with the Declaration of Helsinki.

### Procedure and techniques

**Figure 1:**
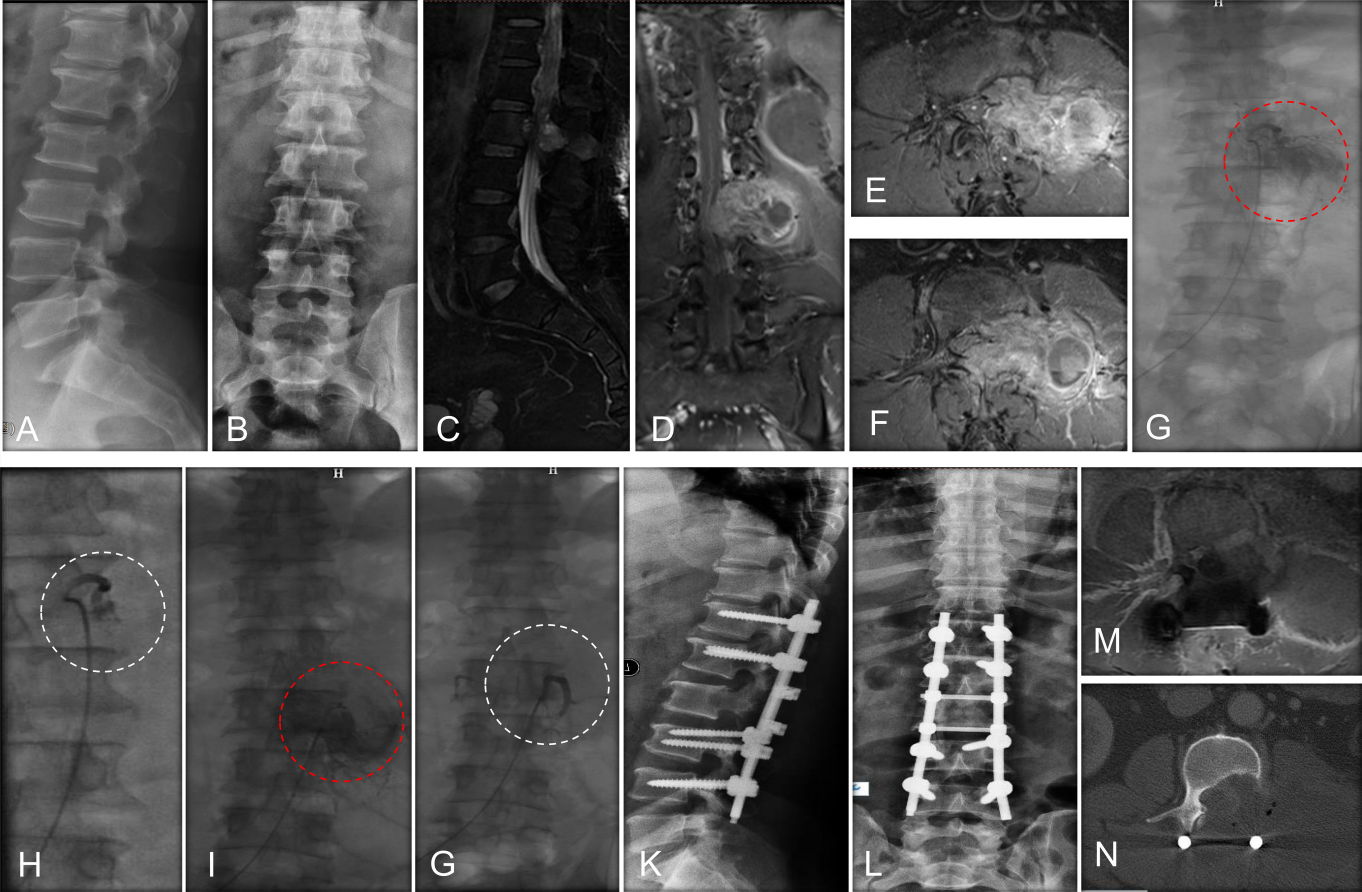
A case report of a MESCC patient who was a 50-year-old man with a histology of renal cancer and treated with preoperative embolization. **(A)** Perioperative lateral X-ray of MESCC; **(B)** Perioperative anteroposterior X-ray showed that pedicle of the vertebral arch disappeared in the left side of L2; **(C)** Preoperative T2-weighted sagittal MRI showing cord compression at L7; **(D)** Preoperative T2-weighted coronal enhanced MRI showing metastatic cancer; **(E)** Preoperative T2-weighted cross enhanced MRI showing metastatic cancer at L1; **(F)** Preoperative T2-weighted cross enhanced MRI showing metastatic cancer at L2; **(G)** Preoperative angiography showed extensive tumor blush from first lumbar artery at left; **(H)** The extensive tumor blush was successfully embolized; **(I)** Preoperative angiography showed extensive tumor blush from the second lumbar artery at left; **(J)** The extensive tumor blush was successfully embolized; **(K)** Lateral radiograph at 1 week after surgery; **(L)** Anteroposterior radiograph at 1 week after surgery; **(M)** MRI of metastatic tumor site at 3 months after surgery, indicating no further tumor progress; **(N)** CT image of metastatic tumor site at 3 months after surgery. Red dotted circle indicates preoperative blush and white dotted circle indicates postoperative blush.

The indication for surgery was neurological deficit, mechanical back pain, and a predicted survival time of more than three months. The main indication of preoperative embolization was to reduce intra-operative bleeding (22), and the aim of embolization was to block the cephalad and caudal segmental arteries. Selection of patients for preoperative embolization was mainly based on the two criteria: (1) preoperative radiography represented hypervascularity; (2) radical surgery was planned. In our institution, preoperative embolization of metastatic spinal tumors was routinely recommended for eligible patients, particularly those with hypervascular tumors, but such surgeries are typically performed in emergencies and limited by the availability of interventional radiologists. In addition, embolization was not done if there was an evidence of major Adamkiewicz artery linked to the tumor vascularization, and Adamkiewicz artery provided blood supply to the spinal cord (23), because embolization of this artery may lead to serious complications, including paralysis, anesthesia, incontinence, and sexual dysfunction. In some cases, embolization was performed partially to preserve the blood supply to the anterior spinal artery. In regional anesthesia, patients received standard endovascular techniques through arterial access to one femoral artery. Selective catheterization and digital subtraction angiography of spinal segmental arteries were performed. Routinely, a 5 F catheter (Cordis) was selectively inserted into thoracic aorta and the corresponding intercostal artery, followed by a 2.6 F catheter (Stride, Japan) being selectively inserted into the branch of intercostal artery. Particles were injected to prevent blood reflux. The interventional radiologist chose the optional embolization material. Polyvinyl alcohol embolization microspheres (500–700 um, Heng Rui, China), gelatin sponge (1000 um, Alicon, China), and microcoils (Cook, Inc, Bloomington, Indiana) were used during embolization. Decompressive surgery was generally conducted within 48 hours after preoperative embolization to avoid the revascularization of the tumor (24). Decompressive surgery was performed by wide laminectomy using the posterior approach, and intralesional excision was conducted as soon as possible in order to prevent massive blood loss. Besides, circumferential decompression was completed as fast as possible, if applicable. Tamponade of the cavity was done using absorbable hemostatic gauze to achieve hemostasis after the removal of tumor tissue. A case report is depicted in **Figure 1**. Intensity-modulated radiotherapy was routinely performed after surgical wound healing among the two groups.

### Baseline characteristics and definitions

**Figure 2:**
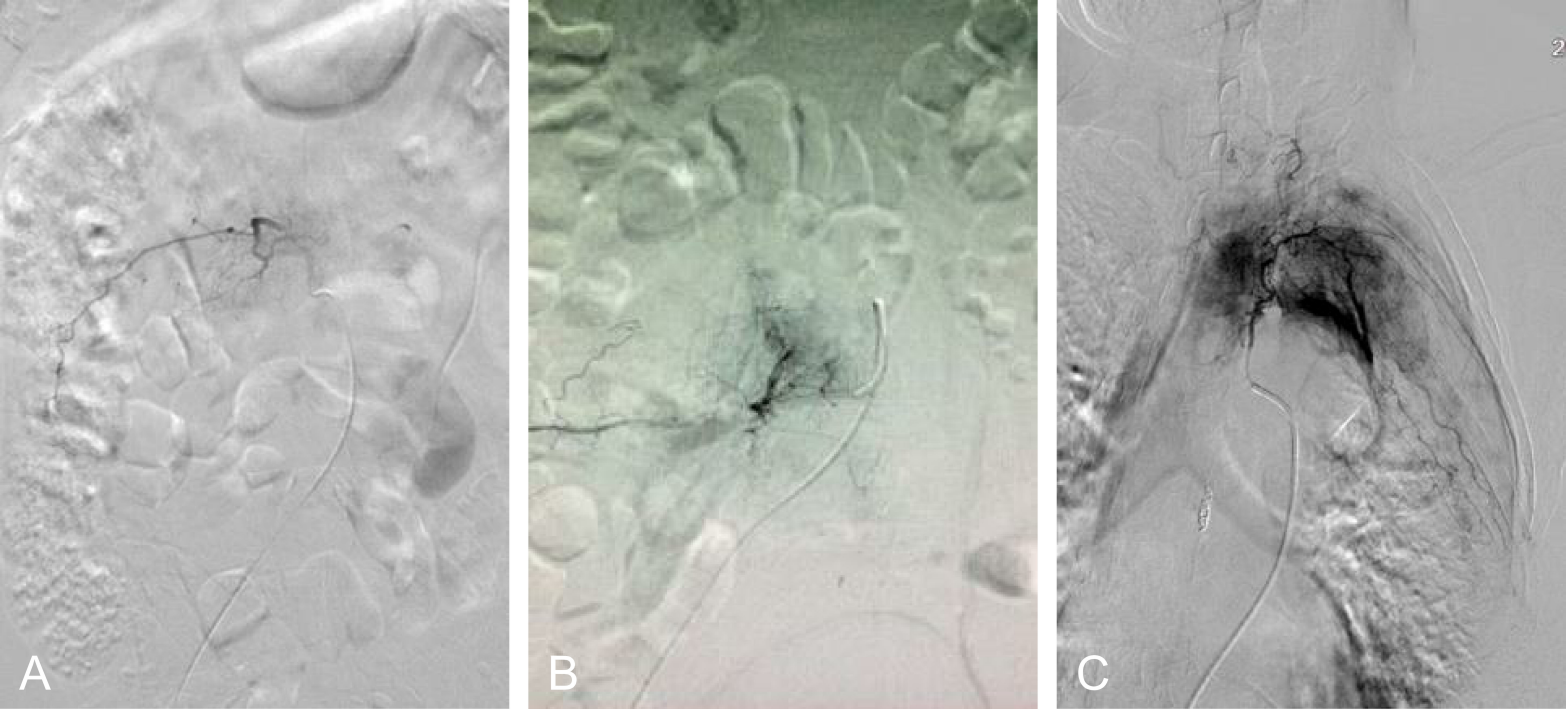
The evaluation of hypervascularity. **(A)** Mild hypervascularity; **(B)** Moderate hypervascularity; **(C)** Severe hypervascularity.

A series of patient’s characteristics, including age, gender, location of MESCC, primary cancer types, preoperative neurological status, spinal instability neoplastic score (SINS), tumor vascularity, preoperative hemoglobin, preoperative international normalized ratio (INR), and preoperative thrombocytes, were collected from the two groups. The preoperative neurological statue was evaluated using ambulatory status (25), and patients with Frankel A, B, and C are unable to walk, while patients with Frankel D and E are ambulatory. The spine instability was evaluated using SINS (26). Before embolization, spinal angiography was used to assess the tumor vascularity in the embolization group by visual evaluation of the intensity of tumor blush (14, 22): mild hypervascularity was defined as no or slightly more prominent than the normal vertebral body blush, moderate hypervascularity was defined as medium tumor blush without early arteriovenous shunting, and severe hypervascularity was defined as intense tumor blush with early arteriovenous shunting. Examples of the degree of vascularity are provided in **Figure 2**. In the control group, hypervascularity was evaluated using tumor histology in terms of previous studies (27). To elaborate, severe hypervascular tumors included hepatocellular cancer, renal cell carcinoma, and thyroid carcinoma (27), moderate hypervascularity included lung cancer, breast cancer, prostate cancer, colon cancer, and nasopharyngeal cancer (27), and mild hypervascularity included others. In addition, subgroup analysis was further performed among patients stratified by severe vs. moderate vs. mild hypervascularity.

### Surgery-related characteristics and definitions

Characteristics evaluated between the two groups included operation time (min), blood loss (ml), number of transfused PRC, and decompressive methods. Operation time was calculated from skin incision to closure, blood loss was determined according to anesthesiologists’ records, and the degree of transfusion was estimated based on the number of used packed red blood cells (RBC). Decompressive methods included circumferential decompression and simple posterior decompression. Circumferential decompression for MESCC was defined as the metastatic tumor being successfully removed around the spinal cord and the complete decompression being achieved. Circumferential decompressive surgery referred to posterolateral transpedicular decompression and tumor resection combined with internal transpedicular screws and rods fixation in the study. Simple posterior decompression was defined as the metastatic tumor being not successfully removed in the anterior spinal cord, and thus the decompression of MESCC was not completely achieved. During surgery, decompression cannot be completely removed mainly due to massive intra-operative blood loss, and thus simple posterior decompression in the study mainly referred to laminectomy and internal transpedicular screws and rods fixation.

### Postoperative outcomes

Postoperative characteristics analyzed in the study included postoperative complication, postoperative neurological status, and postoperative survival outcome. Postoperative neurological status was evaluated ambulatory status one week after surgery (25). The postoperative complications included local and systematic complications due to surgery: local complications included hematoma, infection, or wound dehiscence, and systematic complications included pneumonia, cardiac problem, bedsore or sudden death. Survival time was defined as the overall survival time interval between the operation date and death/or last follow-up.

### Identification of risk factors for affecting blood loss

Identification of risk factors for predicting blood loss were performed in the embolization group after analyze eleven preoperative characteristics, and these characteristics included age, gender (female vs. male), location of MESCC (thoracic spine vs. lumbar spine), ambulatory status (yes vs. no), SINS, tumor vascularity (mild vs. moderate vs. severe), preoperative hemoglobin (mmol/L), preoperative INR, preoperative thrombocytes (×10^9^/L), extent of embolization (partial vs. subtotal vs. total), and time interval of embolization (0–24 h vs. 25–48 h). The extent of embolization was classified into three groups according to the technical success of embolization which was evaluated by visual estimation of tumor blush intensity reduction: partial (<70%), subtotal (70%–90%), and total (>90%) (28).

### Statistical methods

Observational data are reported as mean ± standard deviation (SD). The *t* test, Wilcoxon rank test, and Chi-square test were performed to analyze and compared the patient’s baseline characteristics between two groups. The *t* test, supplied with the Wilcoxon rank test, was used to compare operation time, blood loss, and the number of PRC transfusion. The Chi-square, adjusted continuous Chi-square, and fisher exact test were used to compare postoperative neurological status, complications, and decompressive methods. The log-rank test was used to compare the survival time and Kaplan-Meier method was used to generate the survival curve. Simple and multiple logistic regression models were used to analyze potential characteristics for blood loss. Discrimination of the significant features was evaluated by calculating the area under the receiver operating characteristic curve (AUROC). Calibration was assessed by using the Hosmer-Lemeshow goodness-of-fit test with a P-value of more than 0.05 indicating that there is no evidence of a lack of fit in the selected model. A P value of 0.05 or less was considered statistically significant. Statistical analysis was performed using SAS software (version 9.2), and data visualization was conducted using R programming software (version 4.0)

## Results

### Baseline clinical characteristics

The 138 MESCC patients had a median follow-up of 14.33 months (range: 3.2 to 25.31 months). The mean age at surgery was 58.00 ± 7.43 years in the embolization group and 59.95 ± 8.99 years in the control group (P=0.21). The most common location of MESCC was thoracic vertebra (28/46, 60.9%), followed by lumbar vertebra (18/46, 39.1%) in the embolization group. Similar trend was also observed in the control group for the most common location of MESCC. In the embolization group, 23.9% (11/46) of patients had mild hypervascularity, with 45.7% (21/46) being moderate hypervascularity and 30.4% (14/46) being severe hypervascularity, based on the angiography. The corresponding proportions in the control group were 17.4% (16/92), 50.0% (46/92), and 32.6% (30/92), respectively, in terms of cancer histology. **Table 1** shows more details on baseline clinical characteristics, and it demonstrated that there was no significant difference in the distribution of these clinical characteristics between the two groups.

**Table 1:** Baseline clinical characteristics of the embolization and control groups.

Characteristics	Embolization group (n=46)	Control group (n=92)	P
Age (means ± SD, year)	58.00 ± 7.43	59.95 ± 8.99	0.21
Gender			
Male	21	49	0.40
Female	25	43	
Location of MESCC			
Thoracic spine	28	51	0.54
Lumbar spine	18	41	
Primary cancer types			
Lung cancer	10	20	0.92
Renal cancer	7	17	
Breast cancer	8	18	
Others	21	37	
Preoperative ambulatory status			
No	27	52	0.81
Yes	19	40	
SINS (means ± SD)	7.96 ± 1.86	8.52 ± 2.41	0.17
Tumor vascularity ^a^			
Mild hypervascularity	11	16	0.66
Moderate hypervascularity	21	46	
Severe hypervascularity	14	30	
Preoperative hemoglobin (means ± SD, mmol/L)	7.80 ± 1.01	8.13 ± 1.27	0.12
Preoperative INR (means ± SD)	1.06 ± 0.11	1.04 ± 0.09	0.37
Preoperative thrombocytes (means ± SD, ×10^9^/L)	318.34 ± 72.02	338.27 ± 108.32	0.28

MESCC, metastatic epidural spinal cord compression; SINS, spinal instability neoplastic score; INR, international normalized ratio.

a indicates the tumor vascularity in the control group was evaluated using tumor histology.

### Subgroup analysis of intra-operative characteristics among patients stratified by tumor vascularity

**Figure 3:**
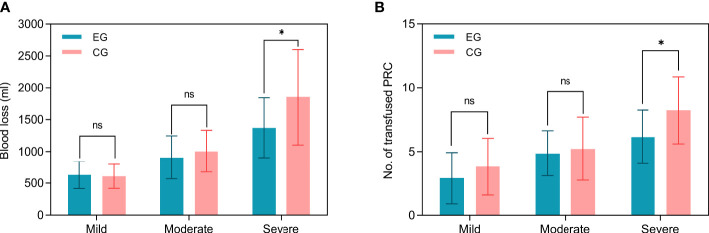
Subgroup analyses of blood loss and number of PRC transfusion among MESCC patients stratified by the degree of hypervascularity. **(A)** Intra-operative blood loss; **(B)** Number of PRC transfusion. EG indicates the embolization group; CG indicating the control group. “ns” indicates no significance, and “*” indicates P<0.05.

Subgroup analysis indicated that mean blood loss was greater in the control group (1852.93 ± 749.31 mL) than in the embolization group especially among patients with severe hypervascularity (1372.14 ± 469.49 mL, **Figure 3A**), and the difference was significant (P=0.02, **Table 2**). However, the blood loss was similar between two groups among patients with mild (P=0.75) and moderate (P=0.67) hypervascularity. In addition, patients with severe hypervascularity in the embolization group also had a significantly lower mean number of transfused PRC as compared with patients in the control group (6.14 ± 2.10 vs. 8.23 ± 2.63 units, P=0.01, **Figure 3B**). However, this trend was also not observed among patients in the mild (P=0.29) and moderate (P=0.96) hypervascularity. With regards to decompressive methods, the embolization group (64.29%, 9/14) had a higher rate of circumferential decompression in comparison to the control group (30.00%, 9/30) among patients with severe hypervascularity (P=0.03), but the rates were similar among patients with mild (P=0.45) and moderate (P=0.54) hypervascularity. Additionally, no subgroup analysis revealed a significant difference in operation time.

**Table 2:** Subgroup analysis of intra-operative and postoperative characteristics among patients stratified by tumor vascularity.

Characteristics	Mild hypervascularity			Moderate hypervascularity			Severe hypervascularity			
	Embolization group(N=11)	Control group(N=16)	P	Embolization group(N=21)	Control group(N=46)	P	Embolization group(N=14)	Control group(N=30)	P
Blood loss (means ± SD, ml)	628.55 ± 213.46	606.81 ± 188.39	0.75	907.00 ± 338.21	1004.63 ± 328.64	0.67	1372.14 ± 469.49	1852.93 ± 749.31	0.02
Number of transfused PRC (means ± SD, unit)	2.91 ± 2.02	3.81 ± 2.23	0.29	4.86 ± 1.77	5.22 ± 2.47	0.96	6.14 ± 2.10	8.23 ± 2.63	0.01
Decompressive methods										
Circumferential decompression	8	9	0.45	8	14	0.54	9	9	0.03
Simple posterior decompression	3	7		13	32		5	21	
Operation time (means ± SD, min)	215.18 ± 49.84	208.42 ± 55.10	0.75	218.24 ± 66.08	223.20 ± 73.15	0.79	229.79 ± 61.53	242.87 ± 72.63	0.71
Ambulatory status										
No	1	3	0.62	3	11	0.57	2	11	0.25
Yes	10	13		18	35		12	19	
Postoperative complication									
Yes	2	2	1.00	4	9	0.96	4	10	0.75
No	9	14		17	37		10	20	

PRC, packed red blood cells; SD, standard deviation.

### Subgroup analysis of postoperative characteristics among patients stratified by tumor vascularity

**Figure 4:**
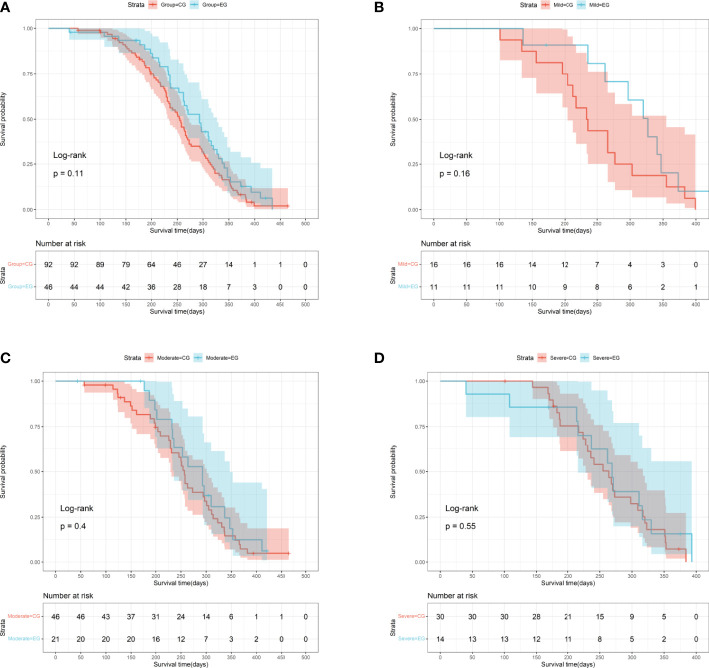
Survival curves for MESCC patients stratified by the presence of preoperative embolization. **(A)** The entire cohort (P=0.11, log-rank test); **(B)** Patients with mild hypervascularity (P=0.16, log-rank test); **(C)** Patients with moderate hypervascularity (P=0.40, log-rank test); **(D)** Patients with severe hypervascularity (P=0.55, log-rank test). EG indicates the embolization group; CG indicating the control group.

This study assessed postoperative ambulatory status, complications, and survival prognoses in relation to postoperative outcomes. Subgroup analysis demonstrated that the influence of preoperative embolization on postoperative ambulatory status was insignificant, although the embolization group had better postoperative functional recovery in comparison to the control group (85.71% (12/14) vs. 63.33% (19/30), P=0.25), in particular among patients with severe hypervascularity (**Table 2**). Regardless of vascularity, the proportions of postoperative complication were similarly distributed between patients with and without preoperative complication (All P≧0.75). Survival outcome was compared between the embolization and control groups, and it showed no significance, with the median survival time of the embolization group being 9.77 (95% CI: 8.43-10.53) months and the control group being 8.50 (95% CI: 7.70-8.93) months (P=0.11, log-rank test, **Figure 4A**). In addition, subgroup analysis of survival outcome was performed in terms of mild (P=0.16, log-rank test, **Figure 4B**), moderate (P=0.40 log-rank test, **Figure 4C**), and severe (P=0.55 log-rank test, **Figure 4D**) hypervascularity, and no significant difference was obtained neither.

### Risk analysis of preoperative clinical characteristics for predicting blood loss

In the univariate analysis of characteristics for blood loss among patients treated with preoperative embolization, significance was found for tumor vascularity (OR=2.98, 95%CI: 1.17-7.57, P=0.02, **Table 3**) and extent of embolization (OR=2.83, 95%CI: 1.00-8.02, P=0.05), whereas no significance was observed for other characteristics (All P>0.05). In the multiply analysis of the risk factors, statistical significance was also observed for tumor vascularity (OR=3.69, 95%CI: 1.30-10.43, P=0.01) and extent of embolization (OR=4.16, 95%CI: 1.10-15.74, P=0.04).

**Table 3:** Univariate and multivariate analysis of characteristics for predicting blood loss among patients treated with preoperative embolization.

Characteristics	Patients (n=46)	Simple logistic regression		Multiple logistic regression	
		OR (95% CI)	P	OR (95% CI)	P
Age (year)	58.00 ± 7.43	1.08 (0.99-1.18)	0.07	Insignificance	
Gender					
Male	21	1.08 (0.33-3.56)	0.90	Insignificance	
Female	25				
Location of MESCC					
Thoracic spine	28	3.12 (0.91-10.79)	0.07	Insignificance	
Lumbar spine	18				
Preoperative ambulatory status					
No	27	1.18 (0.35-3. 94)	0.79	Insignificance	
Yes	19				
SINS	7.96 ± 1.86	1.17 (0.84-1.63)	0.35	Insignificance	
Tumor vascularity					
Mild hypervascularity	11	2.98 (1.17-7.57)	0.02	3.69 (1.30-10.43)	0.01
Moderate hypervascularity	21				
Severe hypervascularity	14				
Preoperative hemoglobin (means ± SD, mmol/L)	7.80 ± 1.01	0.66 (0.36-1.23)	0.19	Insignificance	
Preoperative INR (means ± SD)	1.06 ± 0.11	0.17 (0.10-39.01)	0.52	Insignificance	
Preoperative thrombocytes (means ± SD, 10^9^/L)	318.34 ± 72.02	1.01 (0.99-1.01)	0.30	Insignificance	
Extent of embolization					
Partial	5	2.83 (1.00-8.02)	0.05	4.16 (1.10-15.74)	0.04
Subtotal	17				
Total	24				
Time interval of embolization					
0-24 h	41	7.71 (0.79-75.75)	0.08	Insignificance	
25-48 h	5				

MESCC, metastatic epidural spinal cord compression; SINS, spinal instability neoplastic score; SD, standard deviation; INR, international normalized ratio.

**Figure 5:**
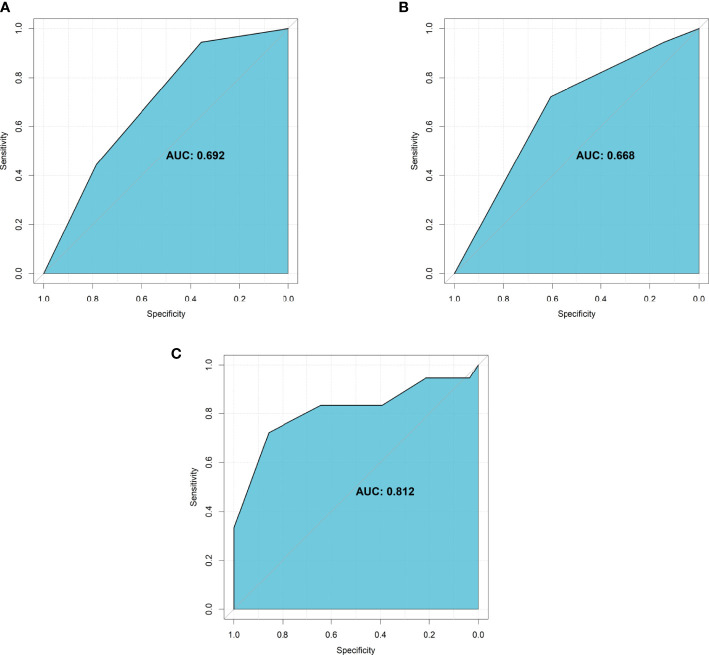
The AUROC of significant characteristics for predicting intra-operative blood loss. **(A)** Tumor vascularity alone; **(B)** Extent of embolization alone; **(C)** The combination of tumor vascularity and extent of embolization.

Evaluation of the two significant factors was conducted using discrimination and calibration. AUROC of the tumor vascularity alone was 0.692 (95% CI: 0.552-0.833) (**Figure 5A**), AUROC of the extent of embolization alone was 0.668 (95% CI: 0.524-0.812) (**Figure 5B**), and AUROC of the tumor vascularity combined with the extent of embolization was 0.812 (95% CI: 0.668-0.957) (**Figure 5C**). In addition, calibration was assessed by using the Hosmer-Lemeshow goodness-of-fit test, and the P-value was 0.32 when the model included tumor vascularity alone, 0.48 when the model included the extent of embolization alone, and 0.06 when the model included the two features.

Additionally, no significant complication was noted in relation to the safety of preoperative embolization. Only four patients suffered from myalgia and one patient had fever because of preoperative embolization, and the symptoms subsided within three days.

## Discussion

Patients with MESCC commonly had decompressive surgical surgery along with postoperative radiotherapy for rapid decompression and local tumor management (4). However, decompressive open surgery may cause significant perioperative blood loss and unfavorable postoperative consequences (5–7). Thus, preoperative embolization has been claimed to reduce intra-operative blood loss among spine metastases patients, particularly those with hypervascular tumors (17, 18), whereas some recent studies have suggested that the blood loss was not different when preoperative embolization was performed among those patients (19, 20). This study examined MESCC patients in great detail using vascularity-based stratification.

In the present study, it demonstrated that patients with severe hypervascular tumor had significant lower blood loss, less number of PRC transfusion, and a higher rate of circumferential decompression, but these effects were not observed among patients with moderate and mild vascular cancers, indicating that preoperative embolization might be an effective therapeutic strategy particularly for MESCC patients with severe hypervascularity. Similar findings were reported in a previous study conducted by Hong et al. (29) and it elucidated that intra-operative blood loss was greater in the non-embolization patients (1988 mL, n=34) than in the embolization patients (1095 ml, n=18) with hypervascular tumor. In a single-blind, randomized controlled clinical study, Clausen et al. (14) also showed that a small reduction of intra-operative blood loss was shown in hypervascular metastases. As for patients with non-hypervascular metastatic spinal tumors, Yoo et al. (16) found that there were no significant differences in intra-operative blood loss, perioperative blood loss, and total blood transfusion between the patients treated with and without preoperative embolization, and the result was consistent with our study.

Furthermore, our study newly proved that patients with severe hypervascular tumor had a significantly higher proportion of circumferential decompression. The reason might be because less intra-operative blood loss provided better surgical views for surgeons, which would definitely facilitate the removal of metastatic tumors. In addition, no significant differences were found in terms of operation time, postoperative ambulatory status, postoperative complication, and survival outcome. The complication rates between the embolization and control groups were similar, which was consistent with other studies (5, 30–32), and the incidence ranged from 15.0% to 35.5%. In a meta-analysis, Gao et al. (32) also found that preoperative embolization had no influence on survival prognosis among spine metastases patients. Regarding the safety of preoperative embolization, some studies has shown that it had the potential risk of hematoma or pseudoaneurysm at the puncture site, arteriovenous fistula, and post-embolization syndrome. The post-embolization syndrome was normally characterized by pain, fever, nausea, myalgia, and general weakness, all of which were possibly originated from tissue infarction due to release of inflammatory mediators and vasoactive substances (14). The symptoms were usually relieved within about 3 days. The frequency of these complications was relatively low, and the number was only 0% to 8.5% (14). In this study, four patients suffer from myalgia and one patient had fever because of preoperative embolization, and the symptoms subsided within 3 days, indicating that preoperative embolization was considered a relatively safe therapeutic approach to treat MESCC patients.

In addition, eleven risk factors were analyzed for intra-operative blood loss among MESCC patients treated with preoperative embolization. Significance was found for tumor vascularity and extent of embolization, while other features showed no significance as risk factors. It indicated the tumor vascularity and the extent of embolization were independent risk factors for predicting blood loss. Other studies showed that more blood loss was found in patients with incomplete embolization (17, 27) and hypervascularity tumor (29). Previous studies also indicated that the location of MESCC and the time interval of embolization both were correlated with intra-operative blood loss. In detail, a decrease in blood loss was related to lumbar localization and the short interval (19, 27). However, other authors found that there was no correlation between the time interval and intra-operative blood loss (33). Thus, this parameter needs further investigation. Evaluation of the two significant features was conducted with the help of discrimination and calibration. It indicated that the tumor vascularity and extent of embolization combined together or alone were useful features for evaluating intra-operative blood loss among MESCC patients.

## Limitations

The study has some constraints. First, it was determined that the study’s bias existed because it was a retrospective analysis and not random. Additionally, some cancer kinds that were thought to have high hypervascularity, like neuroendocrine tumors and hemangiopericytoma instances, were uncommon at our institution. Additionally, several MESCC patients who were paralyzed while admitting the hospital were chosen for urgent surgery without embolization. Although there were some limitations, this study brought great supplements to current literature that preoperative embolization was able to reduce intra-operative blood loss and facilitate surgical removal of metastatic tumors among MESCC patients with severe hypervascularity. Nevertheless, a large prospective study is still warranted.

## Conclusions

Preoperative embolization is an effective and safe method in treating MESCC patients with severe hypervascular tumors in terms of intra-operative blood loss and surgical removal of metastatic tumors. Preoperative tumor vascularity and extent of embolization are independent risk factors for blood loss during surgery. This study implies that MESCC patients with severe hypervascular tumors should be advised to undergo preoperative embolization.

## Data availability statement

The original contributions presented in the study are included in the article/**Supplementary material**. Further inquiries can be directed to the corresponding authors.

## Ethics statement

The studies involving human participants were reviewed and approved by The Ethics Committee Board of the Fourth Medical Center of PLA General Hospital and waived the informed consent from patients since all data were retrospective in nature, and the study was conducted to be in line with the Declaration of Helsinki. Written informed consent for participation was not required for this study in accordance with the national legislation and the institutional requirements.

## Author contributions

BZ, HY, and XZ conceived and designed this study together. BZ, XC, YC, and XS undertook the data analysis, results interpretation and manuscript preparation. XS, ZW, and YL performed supervision. All authors contributed to the article and approved the submitted version.
