# Supplementary figures and images for: Preoperative embolization in the treatment of patients with metastatic epidural spinal cord compression: A retrospective analysis
Source: Front Oncol. 2022 Dec 15;12:1098182. doi: 10.3389/fonc.2022.1098182 (PMC9798328; doi:10.3389/fonc.2022.1098182)

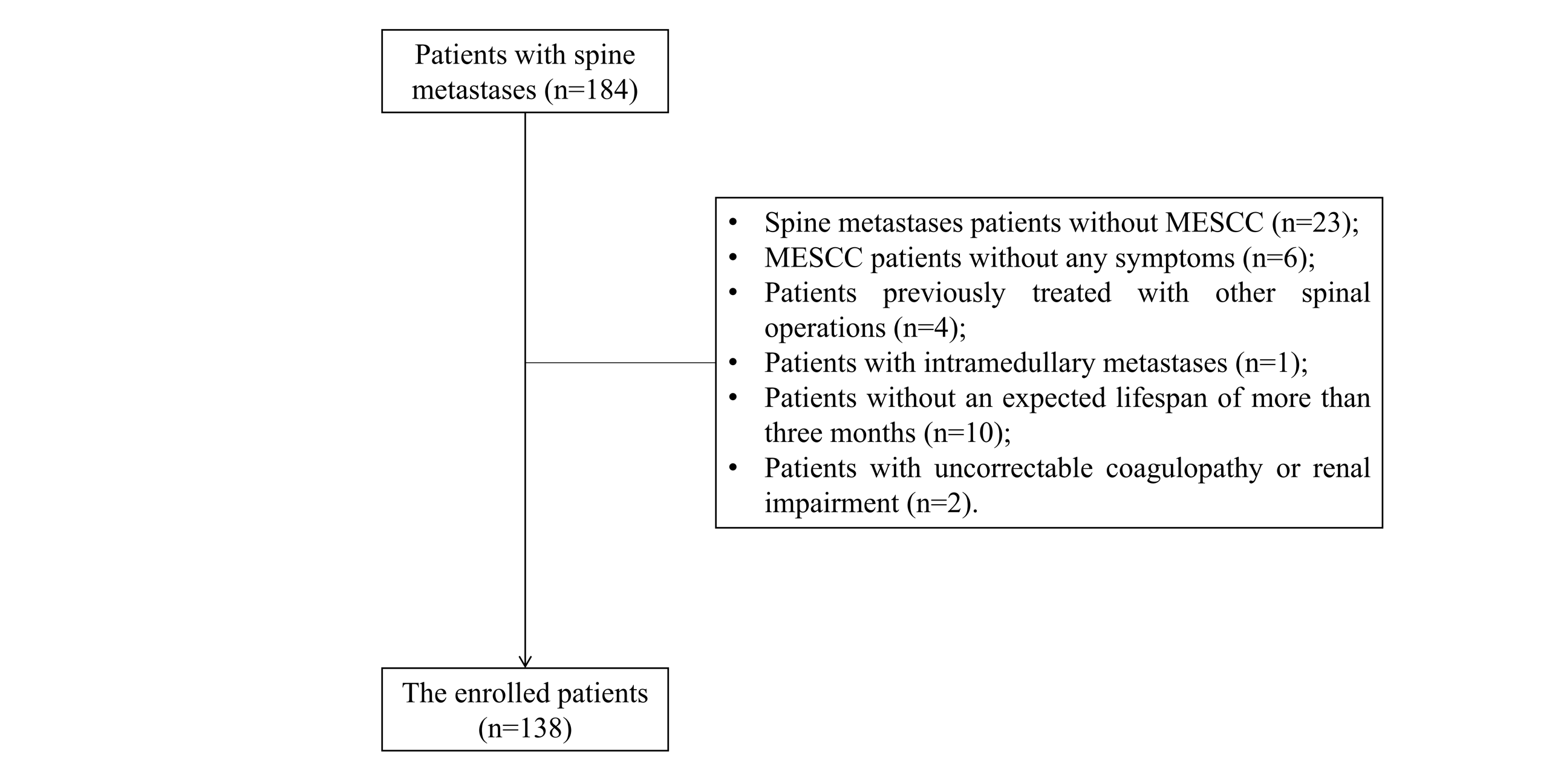

Supplement: Supplementary file 1 [file Image_1.tif]
